# Utilisation du matériel génétique extrait des tests de diagnostic rapide du paludisme en PCR lors du Grand Magal de Touba au Sénégal

**DOI:** 10.48327/mtsi.v5i4.2025.733

**Published:** 2025-08-16

**Authors:** Coumba DIOUF, Ihssane OUADDANE, Georges DIATTA, Mamadou Lamine Bara GOUMBALA, Abdourahmane SOW, Déguène FAM, Mbayang FAYE, Philippe GAUTRET, Cheikh SOKHNA

**Affiliations:** 1 Aix Marseille Université, Institut de recherche pour le développement (IRD), Assistance publique-Hôpitaux de Marseille (AP-HM), Service de santé des armées (SSA), Maladies infectieuses négligées et émergentes au Sud (MINES), Dakar, Sénégal; 2 Aix Marseille Université, IRD, AP-HM, SSA, RITMES, Marseille, France; 3 Institut hospitalo-universitaire (IHU)-Méditerranée Infection, Marseille, France; 4 IRD, Université Cheikh Anta Diop (UCAD), MINES, Dakar, Sénégal

**Keywords:** Paludisme, Test TDR, Sensibilité, Spécificité, Grand Magal de Touba, Mbacké, Touba, Sénégal, Afrique subsaharienne, Malaria, RDT, sensitivity, specificity, Grand Magal of Touba, Mbacke, Touba, Senegal, Sub-Saharan Africa

## Abstract

**Introduction:**

Le Grand Magal de Touba (GMT) est un événement religieux qui attire entre quatre et cinq millions de pèlerins et présente un risque important de transmission de maladies infectieuses dont le paludisme. L’utilisation des tests de diagnostic rapide (TDR) est habituelle lors de cet événement. L’objectif de cette étude était de valider l’utilisation du matériel génétique extrait des TDR pour la réalisation de tests diagnostiques rétrospectifs par PCR.

**Méthode:**

Deux types de TDR Bioline**™** Malaria Ag P. f/Pan (Abbott, USA) et ParaHIT® (Arkray, Inde) utilisés pour la détection de *Plasmodium falciparum* ont été collectés dans huit structures sanitaires du département de Mbacké en 2022 et 2023 pendant le GMT. Une PCR en temps réel (qPCR) a été réalisée après ouverture des tests TDR et extraction de l’ADN/ARN pour confirmer la lecture visuelle des tests antigéniques. Les performances des TDR collectés ont été calculées par rapport à la PCR considérée comme test de référence.

**Résultats:**

2 381 TDR ont été collectés dont 213 TDR positifs (8,9%) d’après la lecture des tests (135 avec les tests Bioline**™** et 78 avec les ParaHIT). En qPCR, 194 échantillons (8,1%) étaient positifs pour *P. falciparum.* Des faux positifs ont été notés dans 39 tests Bioline**™** (2,5%) et 57 tests ParaHIT (7,2%) et des faux négatifs dans 68 tests Bioline**™** (4,3%) et 9 tests ParaHIT (1,1%). Les tests Bioline**™** et ParaHIT avaient des spécificités respectives de 97,3% et 92,5%, des valeurs prédictives positives de 71,1% et 26,9%, et des valeurs prédictives négatives de 95,3% et 98,7% respectivement.

**Conclusion:**

Le matériel génétique extrait des cassettes TDR est utilisable pour réaliser ultérieurement des diagnostics par PCR avec une spécificité et une sensibilité accrue.

## Abstract (en)

**Introduction:**

The Grand Magal of Touba (GMT), a religious event, attracts between four and five million pilgrims and poses a significant risk of infectious disease transmission, including malaria. Rapid diagnostic tests (RDTs) are commonly used during the event. This study aimed to validate the use of genetic material extracted from RDTs for retrospective diagnostic testing by PCR.

**Methods:**

Two types of RDTs were collected from eight health facilities in the Mbacke department in 2022 and 2023 during the GMT: Bioline™ Malaria Ag P.f/Pan (Abbott, USA) and ParaHIT^®^ (Arkray, India) which detects *Plasmodium falciparum.* Real-time PCR (qPCR) was performed after opening the RDTs and extracting DNA/RNA to confirm the visual reading of the antigen tests. The performance of the collected RDTs was calculated relative to the PCR results, which were considered the reference standard.

**Results:**

A total of 2,381 RDTs were collected, 213 of which (8.9%) were positive according to the test readings (135 according to Bioline™ tests, and 78 according to ParaHIT). According to qPCR, 194 samples (8.1%) were positive for *P. falciparum.* There were false positives in 39 (2.5%) Bioline™ Jests and 57 (7.2%) ParaHIT tests, as well as false negatives in 68 (4.3%) Bioline™ tests and 9 (1.1%) ParaHIT^®^ tests. The Bioline™ and ParaHIT^®^ tests were 97.3% and 92.5% specific, respectively. Their positive predictive values were 71.1% and 26.9%, respectively, and their negative predictive values were 95.3% and 98.7%, respectively.

**Conclusion:**

Genetic material extracted from RDT cassettes can be used for subsequent PCR diagnosis with increased specificity and sensitivity.

## Introduction

Le Grand Magal de Touba (GMT) est un événement religieux organisé par la communauté mouride, une confrérie musulmane du Sénégal, célébré le 18 Safar de chaque année selon le calendrier musulman. Il attire entre quatre et cinq millions de pèlerins et présente un risque de transmission de maladies infectieuses, dont la propagation peut être favorisée par la densité de population des participants et la surpopulation des sites les plus fréquentés [3]. Une étude menée de 2018 à 2021 au centre de santé de Mbacké pendant le GMT a montré que sur un total de 141 patients, 45 (31,9%) ont été testés positifs pour au moins un pathogène sanguin, dont 21,3% pour *Plasmodium falciparum,* 5,7% pour *Borrelia* spp. et 5% pour le virus de la dengue [1]. Dans une nouvelle étude, il nous a paru intéressant de vérifier s’il était possible d’utiliser le matériel génétique des tests de diagnostic rapide (TDR) pour confirmer *a posteriori* par PCR le résultat de la lecture initiale.

## Méthode

Pour notre étude, deux types de TDR utilisés pour le diagnostic du paludisme ont été collectées dans huit structures sanitaires du département de Mbacké pendant cinq jours au cours du GMT: le ParaHIT® (Akray Healthcare, Inde) et le Bioline™ Malaria Ag P. f/Pan (Abbott, États-Unis), qui détectent uniquement la protéine HRP2 de *P. falciparum.* Les acides nucléiques (ADN/ARN) ont été extraits des TDR à l’aide de l’instrument KingFisher™ Flex pour effectuer des tests de PCR quantitative en temps réel ciblant l’ARNr 18S de *P. falciparum.* Tous les essais de PCR quantitative en temps réel ont été réalisés à l’aide d’un thermocycleur C1000 Touch™ (Bio-Rad, Hercules, CA, USA) en utilisant la trousse LightCycler® 480 Probes Master kit (Roche Diagnostics, France) conformément aux recommandations du fabricant. Des contrôles négatifs (mélange PCR) et positifs (ADN de souches bactériennes) ont été inclus dans chaque série. Un seuil de cycle (CT) 35 a été utilisé pour les résultats positifs après amplification. La sensibilité, la spécificité, la valeur prédictive positive (VPP) et la valeur prédictive négative (VPN) des TDR du paludisme ont été calculées par rapport à la PCR considérée comme test de référence.

## Résultats

Nous avons collecté 2 381 TDR, dont 1 586 testés par Bioline™ Malaria Ag P. f/Pan et 795 par des tests ParaHIT®. Leur lecture a révélé 213 (8,95%) échantillons positifs (135 pour Bioline™ Malaria Ag P. f/Pan et 78 pour ParaHIT®). En qPCR, 194 (8,15%) étaient positifs (164 pour Bioline™ Malaria Ag P. f/Pan et 30 pour ParaHIT® pour *P. falciparum)* (Tableau I). Les TDR ParaHIT® sont plus sensibles que les Bioline™ Malaria Ag P. f/Pan, mais leur spécificité est légèrement plus faible. Bien que la VPN des deux tests reste correcte, leur VPP est faible, voire médiocre pour le TDR ParaHIT® (Tableau I).

**Tableau I:** Sensibilité, spécificité, valeur prédictive positive et valeur prédictive négative des deux types de TDR *P. falciparum* utilisés

Variables étudiées	Bioline™ *P. falciparum*	ParaHIT® *P. falciparum*
Vrais positifs	96	21
Faux positifs	39	57
Vrais négatifs	1 383	708
Faux négatifs	68	9
Sensibilité	58,5%	26,9%
Spécificité	97,3%	92,6%
Valeur prédictive positive	71,1%	26,9%
Valeur prédictive negative	95,3%	98,7%

## Discussion

Les TDR présentent de nombreux avantages, tels que la rapidité des résultats (5-20 minutes maximum), la facilité d’interprétation et le faible coût. En outre, les TDR du paludisme ont permis de prendre en charge rapidement les patients fébriles, en fournissant un diagnostic probable du paludisme, de sorte que le traitement antipaludique a pu être administré à temps.

Des faux positifs du TDR de *P. falciparum* peuvent être causés par d’autres infections (dengue, toxoplasmose, hépatite C,…) ou des maladies autoimmunes. Des faux négatifs peuvent être dus à des infections pauci-parasitaires, des souches de *P. falciparum* ne sécrétant pas d’HRP2, d’autres espèces plasmodiales, à des erreurs de procédure ou à des tests altérés [2].

Notre étude présente certaines limites. Le délai entre la réalisation des TDR et l’analyse par PCR est long mais il peut être raccourci à l’aide d’une logistique appropriée. Bien que non formellement validée, la technique de récupération des antigènes est confortée par la cohérence des résultats. La différence des résultats des deux TDR entre eux, alors que leurs performances sont similaires, peut s’expliquer par des recrutements de patients et des conditions épidémiologiques du paludisme différents selon les centres de santé.

Néanmoins, l’approche consistant à rechercher directement l’ADN parasitaire par PCR dans le sang collecté sur le TDR lui-même, est originale et incite à réaliser de nombreux tests diagnostiques de fièvres non palustres.

## Éthique

Le protocole a été approuvé par le Comité national d’éthique pour la recherche en santé au Sénégal (SEN17/62), et réalisé conformément aux bonnes pratiques cliniques recommandées par la déclaration d’Helsinki et ses amendements.

## Financement

Cette étude est soutenue par l’Institut hospitalo-universitaire (IHU) Méditerranée Infection, l’Agence nationale de la recherche française dans le cadre du programme des Investissements d’avenir, référence ANR-10-IAHU-03.

## Contribution des auteurs

C. D. a contribué à la conception expérimentale, a rédigé le manuscrit et a réalisé la technique qPCR. I. O. a contribué à la conception expérimentale. G. D. a collecté des échantillons et révisé le manuscrit. M. G. a réalisé la technique qPCR. A. S., D. F. et M. F. ont réalisé la dissection des TDR. P. G. a rédigé le projet original, a contribué à la conception expérimentale, a révisé le manuscrit et a coordonné le travail. C. S. a rédigé le projet original, a contribué à la conception expérimentale et a coordonné le travail.

## Conflits d’intérêts

Les auteurs ne déclarent aucun conflit d’intérêts.
