# Supplementary material for: Use of genetic material extracted from rapid malaria diagnostic tests using PCR during the Grand Magal of Touba in Senegal
Source: Med Trop Sante Int. 2025 Aug 16;5(4):mtsi.v5i4.2025.733. [Article in French] doi: 10.48327/mtsi.v5i4.2025.733 (PMC12948358; doi:10.48327/mtsi.v5i4.2025.733)
Supplement: Related texts

# Research article: Use of genetic material extracted from rapid malaria diagnostic tests using PCR during the Grand Magal of Touba in Senegal

## Introduction

The Grand Magal of Touba (GMT) is an annual religious event organized by the Mouride community, a Muslim brotherhood in Senegal. It is celebrated on the 18^th^ day of Safar according to the Islamic calendar. The event attracts between four and five million pilgrims, which poses a risk of infectious disease transmission due to the density of participants and overcrowding at popular sites [3]. From 2018 to 2021, a study was conducted at the Mbacke health center during the GMT. Out of 141 patients, 45 (31.9%) tested positive for at least one blood pathogen: 21.3% for *Plasmodium falciparum,* 5.7% for *Borrelia* spp., and 5% for the dengue virus [1]. In a new study, we investigated whether polymerase chain reaction (PCR) could be used to confirm initial results from rapid diagnostic tests (RDTs) retrospectively.

## Method

For our study, we collected two types of RDTs used for malaria diagnosis from eight health facilities in the Mbacke department over five days during the GMT. The tests were ParaHIT^®^ (Akray Healthcare, India) and Bioline™ Malaria Ag P.f/Pan (Abbott, USA), which detect only the histidine-rich protein 2 (HRP2) protein of *P. falciparum.* We extracted nucleic acids (DNA/RNA) from the RDTs using a KingFisher™ Flex instrument to perform real-time quantitative PCR tests targeting the 18S rRNA of *P. falciparum.* All real-time quantitative PCR assays were performed using a C1000 Touch™ thermocycler (Bio-Rad, Hercules, CA, USA) with the LightCycler^®^ 480 Probes Master kit (Roche Diagnostics, France), following the manufacturer’s recommendations. Negative controls (PCR mix) and positive controls (DNA from bacterial strains) were included in each run. A cycle threshold (CT) of <35 was used for positive results after amplification. We calculated the sensitivity, specificity, positive predictive value (PPV), and negative predictive value (NPV) of malaria RDTs relative to PCR as the reference test.

## Results

A total of 2,381 RDTs were collected, including 1,586 Bioline™ Malaria Ag P.f/Pan tests and 795 ParaHIT^®^ tests. Testing revealed 213 positive samples (8.95%): 135 for the Bioline™ Malaria Ag P.f/Pan test and 78 for the ParaHIT^®^ test. Of the samples tested by qPCR, 194 (8.15%) were positive (164 for the Bioline™ Malaria Ag P.f./Pan test and 30 for the ParaHIT®test for *P.falciparum)* (Table I). ParaHIT^®^ RDTs are more sensitive than Bioline™ Malaria Ag P.f/Pan, but their specificity is slightly lower. While the NPV of both tests remains acceptable, the PPV is low for both tests and poor for the ParaHIT^®^ RDT (Table I).

**Table I:** Sensitivity, specificity, positive predictive value, and negative predictive value of the two types of *P. falciparum RDTs used*

Variables studied	Bioline™ *P. falciparum*	ParaHIT® *P. falciparum*
True positives	96	21
False positives	39	57
True negatives	1 383	708
False negatives	68	9
Sensitivity	58,5%	26,9%
Specificity	97,3%	92,6%
Positive predictive value	71,1%	26,9%
Negative predictive value	95,3%	98,7%

## Discussion

RDTs have many advantages, such as rapid results (from 5 to 20 minutes maximum), ease of interpretation, and low cost. Additionally, malaria RDTs enable the rapid management of febrile patients by providing a probable malaria diagnosis, allowing for the timely administration of antimalarial treatment.

However, false positives for *P. falciparum* RDTs have been observed and may be caused by other infections, such as dengue, toxoplasmosis, or hepatitis C, etc. as well as autoimmune diseases. False negatives may be caused by low parasite infections, *P. falciparum* strains that do not secrete HRP2, other *Plasmodium* species, procedural errors, or altered tests [2].

Our study has certain limitations. The time between performing RDTs and PCR analysis is lengthy, though it could be reduced with better logistics. Although the antigen recovery technique has not been formally validated, it is supported by consistent results. Despite their similar performance, the difference in results between the two RDTs can be explained by differences in patient recruitment and malaria epidemiological conditions between health centers.

Nevertheless, directly searching for parasitic DNA in blood collected on the RDT itself using PCR is an original approach that encourages performing numerous diagnostic tests for non-malaria fevers.

## Ethics

The protocol was approved by the National Ethics Committee for Health Research in Senegal (SEN17/62) and was conducted in accordance with good clinical practice, as recommended by the Declaration of Helsinki and its amendments.

## Funding

This study is supported by the Institut Hospitalo-Universitaire (IHU) Méditerranée Infection and the French National Research Agency as part of the Investissements d’Avenir program (reference ANR-10-IAHU-03).

## Authors’ contributions

C.D. contributed to the experimental design, wrote the manuscript, and performed the qPCR technique. I.O. contributed to the experimental design. G.D. collected samples and revised the manuscript. M.G. performed the qPCR technique. A.S., D.F., and M.F. performed the RDT dissection. P.G. wrote the original draft, contributed to the experimental design, revised the manuscript, and coordinated the work. C.S. also wrote the original draft, contributed to the experimental design, and coordinated the work.

## Conflicts of interest

The authors declare no conflicts of interest.
